# Antinociceptive and Anti-inflammatory Effects of a Lectin-Like Substance from *Clitoria fairchildiana* R. Howard Seeds

**DOI:** 10.3390/molecules17033277

**Published:** 2012-03-14

**Authors:** Joana Filomena Magalhães Leite, Ana Maria Sampaio Assreuy, Mário Rogério Lima Mota, Pedro Henrique de Souza Ferreira Bringel, Rodrigo Rodrigues e Lacerda, Vinícius de Morais Gomes, João Batista Cajazeiras, Kyria Santiago do Nascimento, Hilzeth de Luna Freire Pessôa, Carlos Alberto de Almeida Gadelha, Plinio Delatorre, Benildo Sousa Cavada, Tatiane Santi-Gadelha

**Affiliations:** 1 Laboratory of Biologically Actives Molecules, Department of Biochemistry and Molecular Biology, Federal University of Ceará, P.O. Box 6043, CEP 60455-970 Fortaleza, Ceará, Brazil; Email: leite.joanna@gmail.com (J.F.M.L.); jcajazeiras@gmail.com (J.B.C.); kyriasantiago@gmail.com (K.S.N.); 2 Institute of Biomedical Sciences, State University of Ceará-Itaperi, CEP 60740-000, Fortaleza, Ceará, Brazil; Email: anassreuy@gmail.com (A.M.S.A.); phbringel@hotmail.com(P.H.S.F.B.); 3 Faculty of Dentistry, Department of Pharmacy, Dentistry and Nursing, Federal University of Ceará, CEP 60430-160, Fortaleza, Ceará, Brazil; Email: mariolmota@yahoo.com.br (M.R.L.M.); 4 Department of Molecular Biology, Federal University of Paraíba, Campus I, CEP 58059-900, João Pessoa, Paraíba, Brazil; Email: rodrigo.jampa@hotmail.com (R.R.L.); vinickjp@gmail.com (V.M.G.); hilzeth57@gmail.com (H.L.F.P.); calbgadelha@gmail.com (C.A.A.G.); pldelatorre@gmail.com (P.D.)

**Keywords:** *Clitoria*, purification, glycoprotein, toxicity, biological activity

## Abstract

Lectins are proteins that have the ability to bind specifically and reversibly to carbohydrates and glycoconjugates, without altering the structure of the glycosyl ligand. They are found in organisms such as viruses, plants and humans, and they have been shown to possess important biological activities. The objective of this study was to purify and characterize lectins in the seeds of *Clitoria fairchildiana*, as well as to verify their biological activities. The results indicated the presence of a lectin (CFAL) in the glutelin acid protein fraction, which agglutinated native rabbit erythrocytes. CFAL was purified by column chromatography ion-exchange, DEAE-Sephacel, which was obtained from a peak of protein retained in the matrix by applying 0.5 M NaCl using the step-wise method. Electrophoretic analysis of this lectin in SDS-PAGE indicated a two band pattern protein molecular mass of approximately 100 and 116 kDa. CFAL proved to be unspecific to all carbohydrates/glycoconjugates in common use for the sugar inhibition test. This lectin showed no significant cytotoxicity to human red blood cells. It was observed that CFAL has anti-inflammatory activity in the paw edema induced by carrageenan model, in which a 64% diminution in edema was observed. Antinociceptive effects were observed for CFAL in the abdominal writhing test (induced by acetic acid), in which increasing doses of the lectin caused reduction in the number of contortions by up to 72%. It was concluded that the purified and characterized lectin from the seeds of *Clitoria fairchildiana* has anti-inflammatory and antinociceptive activity, and is not cytotoxic to human erythrocytes.

## 1. Introduction

Lectins are nonimmune proteins or glycoproteins that have at least one non-catalytic binding site that binds specifically and reversibly to either mono- or oligosaccharides [1,2]. These proteins are distributed in plants, animals and microorganisms [3]. In legumes, they make up about 10% of the total nitrogen of the seeds, thus becoming a potential research product [4]. 

Leguminous lectins show diverse biological activities, such as for paw edema [5], peritoneal leukocyte migration [6], histamine release [7], apoptosis [8], NO production [9], mitosis [10] and cytokine production *in vitro* and *in vivo* [11,12,13]. Additionally, our group demonstrated that plant lectins can display either pro- or anti-inflammatory actions depending on the administration route used, via lectin domain interaction [12,14,15,16]. These effects occur through indirect mechanisms, dependent on macrophage activation by lectins, and possibly leading to the release of neutrophilic chemo- attractive factors [11,17]. Considering the clear association between inflammatory processes and development of pain, a classical sign of inflammation, our group has recently demonstrated the antinociceptive effect of plant lectins [18,19,20] in models of inflammatory pain, so the purification and verification of biological activity for new lectins reveals properties that can be of great importance in biomedical and pharmaceutical research. 

*Clitoria fairchildiana* R. Howard is a legume belonging to the family Fabaceae, subfamily Faboideae, tribe Phaseoleae, and subtribe Clitoriinae, commonly known as Butterfly Pea Tree or “sombrero”. This work describes the isolation, the *in vitro* hemolytic activity and the *in vivo* antinociceptive and antiinflamatory effects of a lectin from *C. fairchildiana*. 

## 2. Results and Discussion

### 2.1. Purification and Chemical Characterization

The protein glutelin fraction showed hemagglutinating activity against rabbit erythrocytes with specific native activity of 326.73 HU mg protein^−1^. This protein fraction is not able to promote hemagglutination of human erythrocytes. These results differ from the seed lectin from *Clitoria ternatea* that agglutinates both native and trypsinised human type B erythrocytes [21]. Unlike the lectin observed in *C. ternatea*, which was inhibited by lactose, galactose and its derivatives, there was no inhibition of hemagglutinating activity by any sugar or glycoprotein tested [21].

The rabbit erythrocytes membrane has a greater diversity in its carbohydrate composition, in other words, various oligosaccharides and glycoconjugates. The distribution of these carbohydrates in the asymmetric membrane occurs in eukaryotic cells [22]. The complexity and organization of these carbohydrates may be promoting the best binding site lectin-carbohydrate arrangement thus enabling hemagglutination. Some lectins have low affinity for monosaccharides or disaccharides due to binding site spatial conformation, and require more complex molecules that favor larger chemical interactions between the lectin and the carbohydrate [23].

This can be observed in the lectin from the seeds of *Eugenia uniflora*, which is nonspecific for any simple carbohydrate, but achieving a considerable rate of inhibition for glycoproteins present in fetal bovine serum, rabbit serum, thyroglobulin, casein and fetuin [24]. Isolectins in *Acacia constricta* and *Phaseolus vulgaris* seeds were inhibited by fetuin and thyroglobulin [25]. As in CFAL, there are also other nonspecific carbohydrate agglutinins, called lectin-like, that have been purified and evaluated for biological functions; the seeds of *Pouteria torta* [26], and BmLec, present in the venom of *Bothrops moojeni* [27]. Santi-Gadelha and collaborators [28] purified and characterized structurally a lectin-like agglutin from seeds of *Acacia farnesiana* found in the albumin fraction which showed anti-inflammatory activity. 

**Figure 1:**
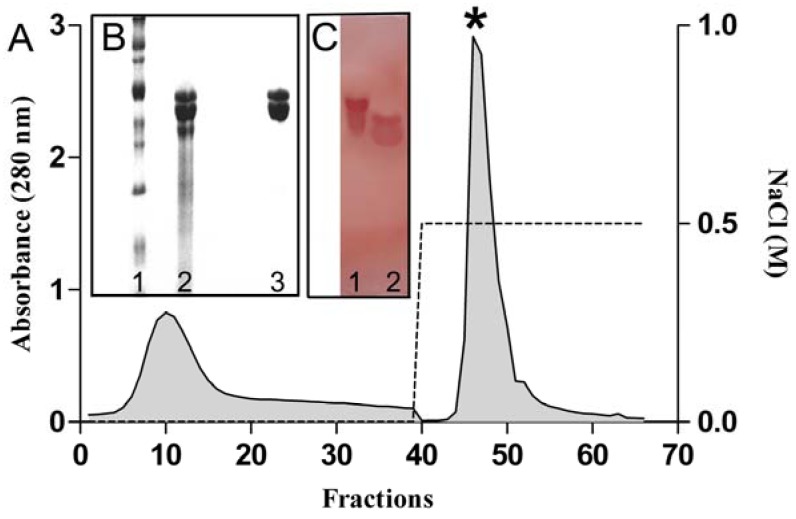
(**A**) Elution profile of the lectin from *C. fairchildiana* on DEAE-Sephacel column (8 × 1.5 cm^2^). Fractions of 2.5 mL were collected and monitored by absorbance at 280 nm. * Peak active; (**B**) SDS-polyacrylamide gel electrophoresis on 12%, lane1: molecular markers: myosin (212 kDa), β-galactosidase (116 kDa), phosphorylase B (97.4 kDa), bovine serum albumin (66.2 kDa), ovalbumin (45 kDa), carbonic anhydrase (31 kDa), trypsin inhibitor soybean (21.4 kDa), and lysozyme at (14.4 kDa), lane 2: fraction acid glutelin, lane 3: peak active; (**C**) SDS-polyacrylamide gel electrophoresis on 12% colored by periodic acid Schiff, lane 1: fetuin swine; lane 2: peak active.

The glutelin acids fraction was subjected to ion-exchange chromatography whereby two peaks were obtained; a peak (PII) was eluted with 0.5 M NaCl. CFAL that was detected only in the PII, being active in promoting agglutination of native rabbit erythrocytes (Figure 1A). The purity of the samples and the apparent molecular weight of CFAL were verified by SDS-PAGE, where there was an electrophoretic pattern of two protein bands with apparent molecular weights of 116 and 100 kDa (Figure 1B). This result differs from that of lectin from *Clitoria ternatea*, which proved to be no longer than one protein subunit of approximately 33.9 kDa, both in native form and reduced form [21].

The lectin of *C. fairchildiana* was shown to be PAS (+) (Figure 1C) thus indicating a glycoprotein nature. The presence of carbohydrates linked to CFAL was measured by the Dubois method [29] showing a concentration of 79 µg/mL carbohydrate. Other lectins such as those from *Bauhinia monandra* [30] and *Luetzelburgia auriculata* [31] are also described as glycoproteins. Sodium metaperiodate is an oxidizing agent capable of promoting ireversible changes in carbohydrates [32], and when treated with sodium metaperiodate, the lectin of *C. fairchildiana* was unable to agglutinate erythrocytes and the probable absence of hemagglutinating activity of lectin in the study may indicate that the carbohydrate portion of the glycoprotein had changed.

### 2.2. Cytotoxic Activity against Human Erythrocytes

The lectin of *C. fairchildiana* induced little hemolytic activity in human erythrocytes; types A (0% in all concentrations), B (3.46% at a concentration of 1,000 µg/mL), and O (2.09%, 1.74%, 1.04% e 5.4%, at concentrations of 1, 10, 100 and 1,000 µg/mL, respectively). Not calculated was the HC_50_ (Hemolytic Media Concentration) since the percentages of hemolysis observed were less than 20%. These data differ from those found by Dresch and collaborators [33] where extracts of *Petromica citrina* and *Acervochalina* sp*.*, showed hemolytic activity for erythrocytes from different animal species. Less than 50% activity after heating at 100 °C, for 10 min in *Acervochalina* sp. extract was observed, suggesting temperature susceptibility. Marine sesquiterpene quinones gave values of HC_50_ ranging from 10 to 10 at 60 µg/mL [34]. 

Lectins can promote different effects on erythrocyte membranes. Some may be able to exert hemolytic activity in rabbit and human erythrocytes, but without promotion of hemagglutination [35], but also exert its cytotoxic activity in some cell types, however other cells are not sensitive to this effect [36].

It is important to investigate the lectins cytotoxic effect on human erythrocytes because, although CFAL not promote hemagglutination of human erythrocytes, could have hemolytic activity on these, this fact is undesirable when it is a molecule with considerable biological action.

### 2.3. Antinociceptive Activity

**Figure 2:**
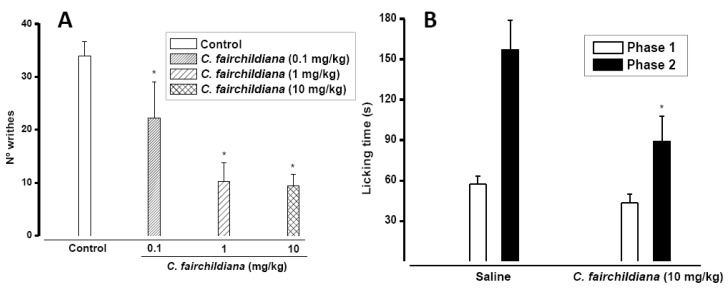
Antinociceptive effect of *C. fairchildiana* lectin. (**A**) *C.fairchildiana* lectin (0.1, 1, 10 mg/kg; i.v.) was injected in mice 30 min before 0.8% acetic acid (v/v; i.p.). Control group received acetic acid only; (**B**) *C. fairchildiana* lectin (10 mg/kg) was injected i.v. 30 min before formalin (1.5% v/v; s.c.). Mean ± S.E.M. (n = 8). * *p* < 0.05 compared to control. ANOVA and Bonferroni test.

In the mice writing test *C. fairchildiana* lectin (0.1, 1, 10 mg/kg) showed significant antinociceptive effects, at all doses tested. The number of writhes was reduced by 35% at 0.1 mg/kg (22 ± 6.81), by 70% at 1 mg/kg (10.25 ± 3.54) and by 72% at 10 mg/kg (9.37 ± 2.27) compared to the control at (34 ± 2.63 writhes) (Figure 2A) Intraperitoneal injection of acetic acid produces nociception via activation of chemosensitive nociceptors [37], or visceral surface irritation, leading to release of inflammatory mediators, such as histamine, bradykinin, prostaglandins and serotonin [38,39]. This antinociceptive effect of C. *fairchildiana* lectin could be associated to anti-inflammatory action.

In the formalin test *C. fairchildiana* lectin (10 mg/kg) showed significant antinociceptive effect, reducing by 43% (89.25 ± 18.64 s) the licking time induced by formalin (157.25 ± 21.87 s), in the inflammatory phase of the test (Figure 2B). The first phase of the formalin test (neurogenic) involves direct stimulation of nociceptors and release of substance P. However, the second phase (inflammatory), is triggered by a combination of stimuli, including inflammation of peripheral tissues and central sensitization [40,41], in which different chemical mediators are involved such as excitatory amino acids, neuropeptides, PGE_2_, nitric oxide, kinins [41,42,43,44,45], serotonin and histamine [46]. It is well known that the actions of analgesic drugs differ in the two phases of the formalin test. Central acting drugs (opiate analgesics) inhibit similarly both phases, while anti-inflammatory drugs (non-opiate analgesics) especially inhibit the second phase [47,48]. Thus, the antinociceptive action of *C. fairchildiana* in the formalin test is in agreement with that obtained in the writing test. 

### 2.4. Anti-inflammatory Activity

**Figure 3:**
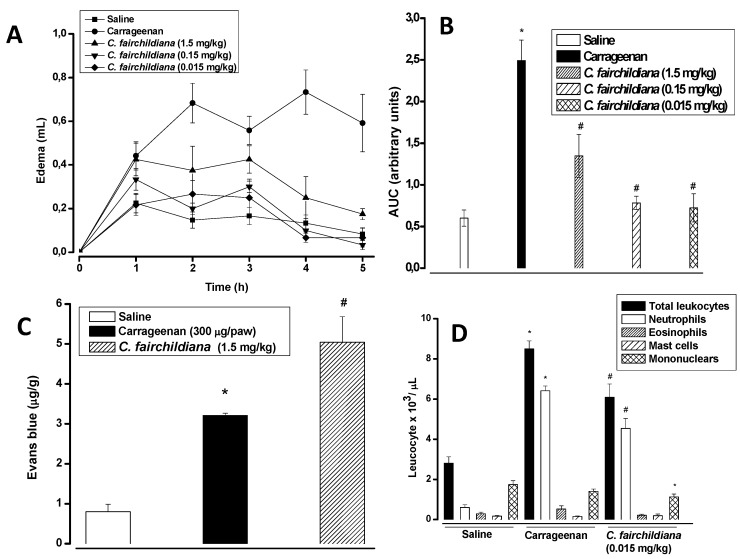
Anti-inflamatory effect of *C. fairchildiana* lectin. *C. fairchildiana* lectin (0.015, 0.15 or 1.5 mg/kg) was injected in rats 30 min before carrageenan at 300 μg/paw or cavity. (**A**) Edema time-course (mL); (**B**) Area under the time-course curve-AUC (arbitrary units); (**C**) Vascular permeability (μg/g Evans blue); (**D**) Leuckocyte migration (×10^3^/μL). Mean ± S.E.M. (n = 6). * *p* < 0.05 compared to saline; # *p* < 0.05 compared to carrageenan. ANOVA and Bonferroni test.

Carrageenan induced intense rat paw edema, that reached maximal levels 4 h after administration (0.73 ± 0.10 mL *versus* saline: 0.13 ± 0.037 mL), and decreased over the subsequent hours. The i.v. treatment of animals with *C. fairchildiana* lectin significantly reduced the edema at all doses, with a maximal inhibition at 0.015 mg/kg (0.067 ± 0.21 mL) 4 h after stimuli. The inhibitory percentages, based on the AUC, were 71%, 68% and 46% at 0.015, 0.15 and 1.5 mg/kg, respectively (Figure 3A and B). However, vascular permeability was not altered by lectin administration at 1.5 mg/kg (Figure 3C).

Carrageenan evokes biphasic edema that lasts up to 6 h: the first two hours are sustained by histamine and serotonin release from mast cells, and the second phase (3–6 h) involves neutrophil infiltrate, and the release of prostaglandin E2, cytokines (mainly interleukin-1β) and NO [49,50]. *C. fairchildiana* lectin showed anti-inflammatory effects via inhibition of the paw edema induced by carrageenan, such effects seem to be associated with the inhibition of neutrophil migration. Carrageenan also elicits increases in vascular permeability and neutrophil migration to the rat peritoneal cavity by an indirect mechanism, via activation of macrophages and mast cells [51].

In the peritonitis model induced by carrageenan (neutrophils: 6,410 ± 238; mononuclears: 1,409 ± 117), *C. fairchildiana* lectin (1.5 mg/kg) decreased either the number of neutrophils (4,535 ± 502 cells), or mononuclear cells (1,133 ± 141 cells) to peritoneal cavities by about 29% and 20%, respectively (Figure 3D). This data corroborates that obtained for carrageenan-induced paw edema, in which the lectin inhibited mainly the edema late phase (rich in leukocyte infiltrate) without interfering with the increase in vascular permeability. 

Experimental studies have demonstrated that inhibition of neutrophil migration reduces hypernociception elicited by different inflammatory stimuli [52,53,54]. *Lonchocarpus sericeus* lectin decreased leukocyte migration and mechanical hypernociception via inhibition of cytokine and chemokine production [18]. Other lectins show antinociceptive activity associated with inhibitory effects on neutrophil migration [19,55]. We speculate that the antinociceptive effect of *C. fairchildiana* lectin could be associated with its inhibitory effect on neutrophil migration.

## 3. Experimental

### 3.1. Plant Material

*Clitoria fairchildiana* seeds were collected in the state of Ceará, Brazil.

### 3.2. Animals

Wistar rats (150–200 g) and Swiss albino mice (25–30 g) were kept in controlled cycles (12/12 h light/dark) with free access to food and water. The experimental protocols were approved by the State University of Ceará-UECE Ethics Committee, (N° 10130208-8/40), Fortaleza, CE-Brazil.

### 3.3. Erythrocytes

Rabbit erythrocytes were obtained from the Federal University of Paraiba, in Brazil, and human blood was obtained from healthy donors at the Hematology Center of Paraiba.

### 3.4. Purification of *C. fairchildiana* Lectin

*Clitoria fairchildiana* flour was de-lipidated with hexane, extracted with 0.15 M NaCl, and fractioned, and after centrifuging at 9,000 rpm (4 °C) we obtained the albumins and globulins. To the centrifuged precipitate was added 0.1 M HCl (100 mL) and it was subjected to 2 h of extraction, centrifuged at 9,000 rpm (4 °C), and the supernatant fraction, called the glutelin fraction, was dialyzed and lyophilized for subsequent analyses. The glutelin fraction (10 mg) was applied to a ion exchange column of DEAE-Sephacel and equlibrated with 0.025 M Tris-HCl (pH 7.6). The material not retained (Peak I) was eluted with column equilibration buffer, and the lectin retained on the column (Peak II) was eluted with 0.5 M NaCl in 0.025 M Tris-HCl (pH 7.6) to yield 4.4 mg of CFAL.

The quantification of soluble proteins was assayed by Bradford [56] using bovine serum albumin as the standard, and determination of soluble carbohydrates was determined by the Dubois method [29] using glucose as the standard.

### 3.6. Hemagglutinating Activity

Hemagglutinating activity was determined in tubes by serial dilution. All tubes in the series received 100 μL of 0.1 M Tris-HCl buffer (pH 7.6) containing 0.15 M NaCl. In the first tube of the series, 100 μL of that which was obtained in step 3.4 (fraction and peaks) was added. The second tube received 100 μL from the mixture of the first and so on until the last tube accumulated 200 μL, and was left out of the assay. Afterwards, 100 μL of 3% rabbit and human erythrocyte suspension in the same buffer was added to each tube. Hemagglutination was determined after 1h of incubation at 37 °C. Hemagglutinating activity was expressed as a titer, namely the reciprocal of the highest dilution that gave a positive result. 

### 3.7. Sugar Specificity

The lectin sugar specificity determination was tested by comparing the inhibitory activity of sugars and glycoproteins on hemagglutination. The initial concentrations of carbohydrates and glycoproteins were 0.1 M and 5 mg/mL, respectively. The carbohydrates used included D-glucose, D-galactose, D-mannose, *N*-acetyl-D-glucosamine, *N*-acetyl-D-galactosamine, L-fucose, lactose, α-methylmannoside, fucoidan, carrageenan, mucin and fetuin. Results were expressed as the minimum sugar or glycoprotein concentration required to inhibit hemagglutination.

### 3.8. Electrophoresis Polyacrylamide Gel-SDS (SDS-PAGE)

The SDS-polyacrylamide gel electrophoresis was carried out on slabs of 12% polyacrylamide gel and 3% stacking gel and run at 20 mA for 4 h Laemmli [57]. The molecular markers used were myosin (212 kDa), β-galactosidase (116 kDa), phosphorylase B (97.4 kDa), bovine serum albumin (66.2 kDa), ovalbumin (45 kDa), carbonic anhydrase (31 kDa), trypsin inhibitor soybean (21.4 kDa), and lysozyme at (14.4 kDa). The protein bands were viewed by staining with coomassie brilliant blue R 250. To identify glycoproteins, the polyacrylamide electrophoresis was colored by periodic Schiff acid (PAS) in the second method described by Kapitany and Zabrouski [58] using swine fetuin as the standard.

### 3.9. Action of Sodium Metaperiodate Oxidizing Agent

An aliquot of lectin was diluted in sodium metaperiodate (10 mM), diluted in 10 mM sodium acetate buffer solution (pH 5.5) and subsequently incubated for 10 min at room temperature while being protected from light. The solution was dialyzed against 0.1 M Tris-HCl buffer (pH 7.6) with 0.5 M NaCl for 12 h. The haemagglutinating activity test was performed afterwards to verify the integrity of the lectin.

### 3.10. Nociceptive Models in Mice

#### 3.10.1. Writing Test

Acetic acid (0.8%; v/v; 0.1 mL/10 g body weight) was intraperitoneally injected (i.p.) in the mice. Ten min later the number of abdominal constrictions (writhes) was recorded at 20 min [59]. Animals were treated intravenously (i.v.) with *C. fairchildiana* lectin (0.1, 1.0, 10 mg/kg) or sterile saline (0.9% w/v NaCl) 30 min before the acetic acid.

#### 3.10.2. Formalin Test

Twenty microliters of 1.5% formalin (v/v in distilled water) were injected by subcutaneous route (s.c.) in the dorsal surface of the mice right-hind paws. Immediately after formalin, animals were individually placed in a glass observation chamber with a transparent floor, beneath which a mirror was mounted at a 45 °C angle to allow clear observation of animal paws. The time (in seconds) that animals spent licking the injected paws was recorded during the first 5 min of formalin injection (1st phase, corresponding to direct chemical stimulation of nociceptors), and from 15 to 30 min (2nd phase, involving the release of inflammatory mediators) [47]. The animals received sterile saline (0.1 mL, i.v.), or *C. fairchildiana* lectin (10 mg/kg, i.v.) 30 min before the formalin.

### 3.11. Inflammatory Models in Rats

#### 3.11.1. Paw Edema

Paw edema was induced by intraplantar s.c. injection of 0.1 mL of carrageenan (300 µg/paw). Paw volume displacement was measured, with a hydroplethysmometer (PanLabs, Barcelona-Spain) apparatus, immediately before stimuli (zero time), and at selected time intervals thereafter (1, 2–5 h). Control animals received sterile saline s.c. instead of carrageenan. Results were expressed as the increase in paw volume (mL), calculated by subtraction of the basal volume, measured at zero time, or as the area under the time-course curve-AUC (arbitrary units), using the trapezoidal rule, and compared to control animals injected with the same volume of sterile saline [60].

Vascular permeability was evaluated 1 h after i.v. administration of Evans’ blue (25 mg/kg), time in which the animals were sacrificed by cervical dislocation in the fifth hour after the beginning of experiment. Paws were sectioned, weighed and incubated in formamide for 72 h at 37 °C. The extracted dye was estimated at A_600 nm_ (µg Evans’ blue/g tissue) [61]. The animals were treated (i.v.) with *C. fairchildiana* lectin 30 min before carrageenan at 0.015, 0.15, and 1.5 mg/kg for evaluation of edema, and at 1.5 mg/kg for evaluation of vascular permeability.

#### 3.11.2. Peritonitis

Peritonitis was induced by i.p. injection of 1 mL carrageenan (300 μg), or sterile saline (0.9% w/v NaCl). Four hours later, animals were sacrificed and the peritoneal cavity washed with 10 mL of saline containing 5 IU/mL heparin. Peritoneal fluid was recovered for total and differential leukocyte (neutrophil, eosinophil, mast cell, mononuclear) counts. Results were expressed as number of cells × 10^3^/mL of peritoneal fluid [62]. Animals were treated, i.v. with *C. fairchildiana* lectin (1.5 mg/kg) or sterile saline (0.9%), 30 min before stimuli. 

### 3.12. Assessment of the Potential Cytotoxicity Assay of Lectin from *C. fairchildiana* in Human Erythrocytes

Different concentrations of lectin (1, 10, 100 e 1,000 µg/mL) were added to a 2 mL suspension of human erythrocytes (A, B e O) at 0.5%. As negative control we used 0.15 M NaCl, and as positive control we used Triton X-100. After incubating for 1 h at 25 °C, slow and steady agitation (100 rpm), hemolysis was quantified by spectrophotometry at 540 nm [63]. The results were expressed as an average ± standard deviation of three independent experiments.

### 3.13. Statistical Analysis

Results were presented as the mean ± S.E.M. of animals (n = 6–8) and statistical differences were detected by variance analysis (ANOVA), and followed by the Bonferroni correction test, *p* < 0.05 was considered significant.

## 4. Conclusions

A lectin from *C. fairchildiana* was purified and shown to be a glycoprotein with an electrophoretic pattern consisting of two bands with molecular weights of approximately 100 e 116 kDa. The characteristic lectin was confirmed to be capable of binding native rabbit erythrocytes. This lectin has antinociceptive action, probably through a peripheral mechanism, and anti-inflammatory action, associated with an inhibitory mechanism on neutrophil migration. These results point out the need for investigations into the possible use of *C. fairchildiana* lectin as a prototype in the development of new drugs to treat inflammatory pain.
